# Validation of an Electronic Health Record-Based Suicide Risk Prediction Modeling Approach Across Multiple Health Care Systems

**DOI:** 10.1001/jamanetworkopen.2020.1262

**Published:** 2020-03-02

**Authors:** Yuval Barak-Corren, Victor M. Castro, Matthew K. Nock, Kenneth D. Mandl, Emily M. Madsen, Ashley Seiger, William G. Adams, R. Joseph Applegate, Elmer V. Bernstam, Jeffrey G. Klann, Ellen P. McCarthy, Shawn N. Murphy, Marc Natter, Brian Ostasiewski, Nandan Patibandla, Gary E. Rosenthal, George S. Silva, Kun Wei, Griffin M. Weber, Sarah R. Weiler, Ben Y. Reis, Jordan W. Smoller

**Affiliations:** Computational Health Informatics Program, Boston Children’s Hospital, Boston, Massachusetts; Partners Research Information Science and Computing, Boston, Massachusetts; Department of Psychology, Harvard University, Cambridge, Massachusetts; Computational Health Informatics Program, Boston Children’s Hospital, Boston, Massachusetts; Department of Biomedical Informatics, Harvard Medical School, Boston, Massachusetts; Psychiatric and Neurodevelopmental Genetics Unit, Center for Genomic Medicine, Massachusetts General Hospital, Boston, Massachusetts; Psychiatric and Neurodevelopmental Genetics Unit, Center for Genomic Medicine, Massachusetts General Hospital, Boston, Massachusetts; Department of Pediatrics, Boston Medical Center, Boston University School of Medicine, Boston, Massachusetts; School of Biomedical Informatics, The University of Texas Health Science Center at Houston, Houston; School of Biomedical Informatics, The University of Texas Health Science Center at Houston, Houston; McGovern Medical School, Division of General Internal Medicine, The University of Texas Health Science Center at Houston, Houston; Partners Research Information Science and Computing, Boston, Massachusetts; Department of Medicine, Beth Israel Deaconess Medical Center, Boston, Massachusetts; Partners Research Information Science and Computing, Boston, Massachusetts; Computational Health Informatics Program, Boston Children’s Hospital, Boston, Massachusetts; Clinical and Translational Science Institute, Wake Forest School of Medicine, Winston-Salem, North Carolina; Computational Health Informatics Program, Boston Children’s Hospital, Boston, Massachusetts; Department of Internal Medicine, Wake Forest School of Medicine, Winston-Salem, North Carolina; Department of Medicine, Beth Israel Deaconess Medical Center, Boston, Massachusetts; Clinical and Translational Science Institute, Wake Forest School of Medicine, Winston-Salem, North Carolina; Department of Biomedical Informatics, Harvard Medical School, Boston, Massachusetts ;Department of Medicine, Beth Israel Deaconess Medical Center, Boston, Massachusetts; Department of Biomedical Informatics, Harvard Medical School, Boston, Massachusetts; Computational Health Informatics Program, Boston Children’s Hospital, Boston, Massachusetts; Psychiatric and Neurodevelopmental Genetics Unit, Center for Genomic Medicine, Massachusetts General Hospital, Boston, Massachusetts

## Abstract

**IMPORTANCE:**

Suicide is a leading cause of mortality, with suicide-related deaths increasing in recent years. Automated methods for individualized risk prediction have great potential to address this growing public health threat. To facilitate their adoption, they must first be validated across diverse health care settings.

**OBJECTIVE:**

To evaluate the generalizability and cross-site performance of a risk prediction method using readily available structured data from electronic health records in predicting incident suicide attempts across multiple, independent, US health care systems.

**DESIGN, SETTING, AND PARTICIPANTS:**

For this prognostic study, data were extracted from longitudinal electronic health record data comprising *International Classification of Diseases, Ninth Revision* diagnoses, laboratory test results, procedures codes, and medications for more than 3.7 million patients from 5 independent health care systems participating in the Accessible Research Commons for Health network. Across sites, 6 to 17 years’worth of data were available, up to 2018. Outcomes were defined by *International Classification of Diseases, Ninth Revision* codes reflecting incident suicide attempts (with positive predictive value >0.70 according to expert clinician medical record review). Models were trained using naive Bayes classifiers in each of the 5 systems. Models were cross-validated in independent data sets at each site, and performance metrics were calculated. Data analysis was performed from November 2017 to August 2019.

**MAIN OUTCOMES AND MEASURES:**

The primary outcome was suicide attempt as defined by a previously validated case definition using *International Classification of Diseases, Ninth Revision* codes. The accuracy and timeliness of the prediction were measured at each site.

**RESULTS:**

Across the 5 health care systems, of the 3 714 105 patients (2 130 454 female [57.2%]) included in the analysis, 39 162 cases (1.1%) were identified. Predictive features varied by site but, as expected, the most common predictors reflected mental health conditions (eg, borderline personality disorder, with odds ratios of 8.1-12.9, and bipolar disorder, with odds ratios of 0.9-9.1) and substance use disorders (eg, drug withdrawal syndrome, with odds ratios of 7.0-12.9). Despite variation in geographical location, demographic characteristics, and population health characteristics, model performance was similar across sites, with areas under the curve ranging from 0.71 (95% CI, 0.70-0.72) to 0.76 (95% CI, 0.75-0.77). Across sites, at a specificity of 90%, the models detected a mean of 38% of cases a mean of 2.1 years in advance.

**CONCLUSIONS AND RELEVANCE:**

Across 5 diverse health care systems, a computationally efficient approach leveraging the full spectrum of structured electronic health record data was able to detect the risk of suicidal behavior in unselected patients. This approach could facilitate the development of clinical decision support tools that inform risk reduction interventions.

## Introduction

Suicide is the tenth leading cause of death in the US^1^ and the second most common cause of death among young US individuals.^2^ This important public health threat is increasing, because rates of death by suicide increased by 30% between 2000 and 2016.^3^ A substantially larger number of nonfatal suicide attempts occur annually, estimated to be 1.3 million cases in 2016.^4^ Early and accurate identification of individuals at elevated risk for suicide attempts and death is critical for developing and implementing effective strategies to prevent suicidal behavior. Although numerous factors, such as depression, substance abuse, and access to firearms, have been associated with suicide risk, they have not proven useful for individualized risk prediction. Indeed, a comprehensive meta-analysis^5^ of 50 years of research on longitudinal prediction of suicidal thoughts and behaviors concluded that the predictive performance of available risk factor profiles is only slightly better than chance and has not improved over the last half century.

In recent years, 2 developments have created new opportunities to improve this dire state of affairs. First, vast and rapidly growing longitudinal data resources capturing real-world health data and outcomes have become widely available through the adoption of electronic health records (EHRs) in most clinical settings. Second, a growing number of statistical and machine learning methods have been used successfully to produce prediction algorithms that leverage highdimensional data sources (ie, big data). The application of these methods to EHR data provides a means of generating predictions from a far more complex set of information than any human clinician could reasonably manage. Several research groups have begun to apply these approaches to the prediction of suicidal behavior in health care settings.^6-8^ Given that most suicide decedents have contact with health care practitioners within 1 month to 1 year of their deaths,^9,10^ these settings provide crucial opportunities for risk detection and prevention. In addition, most of those who die by suicide do not have a known prior mental health condition,^11^ underscoring the value of examining a broad range of potential predictors beyond traditional psychiatric risk factors.

We recently reported the development and validation of an EHR-based risk prediction model for suicidal behavior in a large health care system (Partners HealthCare) using data from more than 1.7 million patients.^6^ At a specificity of 90%, the model detected 45% of incident suicide attempts a mean of 3 to 4 years in advance, with an area under the receiver operating curve (AUC) of 0.77.^6^

Here, we report the performance of our modeling strategy across 5 large health systems comprising data on more than 3.7 million patients. The goal of this study was to test whether an EHR-based suicide risk modeling approach can accurately predict the risk of a first (index) suicide attempt across diverse health care systems.

## Methods

This research was approved by the Partners HealthCare Human Research Committee and by the other participating sites through a SMART institutional review board reliance agreement. A waiver of informed consent was granted because the study used only deidentified data and summary statistics. This report follows the Standards for Reporting of Diagnostic Accuracy (STARD) reporting guideline.

### Case Definition and Inclusion Criteria

Details of the development of our EHR-based case definitions and risk model for suicidal behavior in the Partners HealthCare system are reported elsewhere.^6^ We previously identified candidate *International Classification of Diseases, Ninth Revision (ICD-9)* codes that are likely to capture suicide attempts.^6^ Three expert clinicians reviewed more than 2700 clinical notes from 520 patients at Partners HealthCare and identified a set of *ICD-9* codes that reliably captured suicide attempts, with a positive predictive value (PPV) of greater than 0.70: E95*, 965*, 967*, 969*, and 881*. Cases were defined as individuals having at least 1 of these codes in their EHR.

In the present analyses, we retrained our Partners HealthCare model using an expanded data set. We included all inpatient and outpatient visits occurring between 1998 and 2015 (inclusive) at Massachusetts General Hospital, Brigham and Women’s Hospital, and McLean Hospital (all in Boston, Massachusetts). We included patients with 3 or more visits, 30 days or more between the first and last visits, and the existence of records after age 10 years and before age 90 years. Data were collected up to but not including the first suicidal event for the case patients and for all observed time periods for noncases; as a result, cases without recorded data before their first suicidal event were excluded from our analysis. After applying the aforementioned definitions, we identified 25 730 cases and 1905 942 noncases with sufficient data for inclusion in model training.

To develop our updated suicide-prediction algorithm, we divided our cohort into equally sized training and testing (validation) subsets. The model incorporated information on demographic characteristics, diagnostic codes, laboratory test results (normal, low, or high), and prescribed medications (true or false values). To simplify the modeling procedure, each predictor was counted only at its first occurrence and was represented as a binary yes or no variable.

Similar to our previous work,^6^ we developed a suicide-risk prediction algorithm using the naive Bayes classifier (NBC) approach, a supervised learning method that has been shown to be well-suited for clinical decision support and classification tasks^12^ and has the advantage of being highly scalable and interpretable. Each independent input variable in the training data set (eg, diagnoses and medications) was assigned a partial risk score on the basis of the ratio of its prevalence among cases vs noncases. The score was calculated on a logarithmic scale; thus, negative scores were protective (ie, not associated with suicidal behavior), and positive scores were adverse (ie, with higher prevalence among cases).

### Implementation of the Risk Modeling Approach in Multiple Independent Health Care Systems

Validation of the modeling approach was performed at health centers participating in the Accessible Research Commons for Health (ARCH) network.^13^ Through a federated network model, ARCH provides access to EHR data and patient populations across 10 health systems that have entered into a consortium agreement.^13^ The data at each site are stored in open source data analytic nodes in a common data model (i2b2; i2b2 tranSMART Foundation).^14^ A federated query format^15^ enables ready identification of cohorts using Boolean logic or computable phenotypes. Each site in the ARCH network stores EHR data using a standard i2b2 schema and maps their local diagnosis, medication, procedure, and laboratory test information into a normalized ARCH ontology based on *ICD-9, Current Procedural Terminology Version 4,* Logical Observation Identifier Names and Codes, and RxNorm codes.

Overall, 6 sites were considered for inclusion in the study: (1) Partners HealthCare System, Boston; (2) Boston Medical Center (BMC), a 567-bed academic hospital, Boston; (3) Wake Forest Medical Center (WF), including 1 academic hospital (WF Baptist Medical Center), 1 children’s hospital (Brenner Children’s Hospital), 4 community hospitals, and 232 outpatient clinics, Winston-Salem, North Carolina; (4) The University of Texas Health Science Center at Houston, which includes data from the University of Texas Physicians network or clinics, including ambulatory clinics and some surgical centers; (5) Beth Israel Deaconess Medical Center (BIDMC), including 1 academic hospital with 673 licensed beds, including 493 medical or surgical beds, 77 critical care beds, and 62 obstetrics and gynecology beds (both inpatient and outpatient data), Boston; and (6) Boston Children’s Hospital (BCH), a 404-bed comprehensive center for pediatric health care, with approximately 25 000 inpatient admissions each year and 557 000 visits annually (including ambulatory care), Boston. From all sites combined, we received a total of 71 health system-years of data, comprising Partners with 17 years of data, BMC with 17 years, WF with 10 years, University of Texas with 13 years, BIDMC with 6 years, and BCH with 8 years of data. Data from BIDMC were excluded from the study because of significant data incompleteness issues in the shared BIDMC ARCH data repository and because of a BIDMC transition from *ICD-9* to *International Statistical Classification of Diseases and Related Health Problems, Tenth Revision* during the study period, which prevented application of a consistent case definition over the entire study period.

Race and ethnicity data were self-reported by each patient at the time of hospital registration. These data are available across ARCH sites and were included to account for differences in outcomes by race/ethnicity categories.

**Figure 1.**
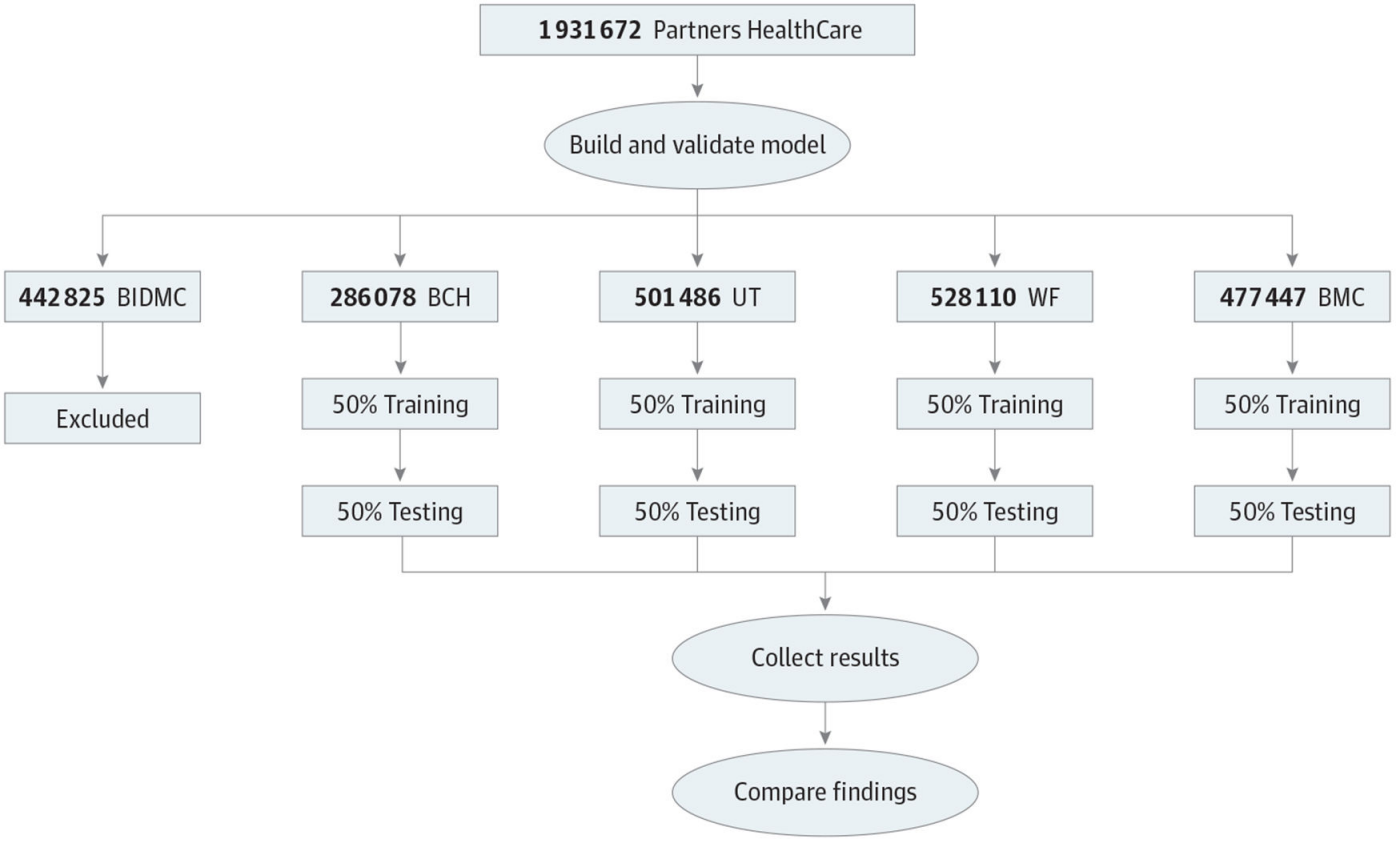
Flowchart of Study Design Model was built using Partners Healthcare data, and then the model’s code was sent to all sites where it was recalibrated using local training subcohorts. The model was then validated on local validation subcohorts and the results were sent back to Partners. BCH indicates Boston Children’s Hospital; BIDMC, Beth Israel Deaconess Medical Center; BMC, Boston Medical Center; UT, The University of Texas Health Science Center at Houston; and WF, Wake Forest Medical Center.

To implement our model development process across sites, each of the collaborating sites was sent SQL code for querying their local i2b2 database. The SQL code was used to extract the required data sets from the local databases into flat files that could be used to run the predictive model. Each ARCH site was also sent modeling code for the NBC, written in R,^16^ which was developed and validated using Partners data.^6^ At each site, we randomly divided the site’s data into 50% training and 50% validation sets. The model was retrained on the training set at each site, resulting in site-specific risk scores (Figure 1). Thus, all the sites used the same R code to run the prediction model, but a separate set of coefficients was derived from the training set at each of the sites. The R and SQL codes have been published elsewhere.^17^

### Statistical Analysis

At each site, we evaluated model performance using the randomly selected 50% validation set. This included all available information on the noncases and all information up to the first (index) suicide attempt for cases. Each concept code (eg, laboratory test, medication, diagnosis, procedure code, or demographic feature) was matched with its partial risk score that was calculated using the training set at that site. We then calculated the cumulative risk score over time for each individual, summing these partial risk scores in the order that they were recorded. The maximal score for each patient was used for model evaluation. We set model thresholds for detection to achieve a desired level of specificity (eg, the 90% percentile of all noncases’ maximal score) and then measured the resulting sensitivity. To measure the timeliness of predictions for each individual identified as a case, we looked for the first point in time in which the individual’s cumulative score surpassed the risk score threshold that achieved the desired level of specificity in the training set and measured the amount of time until the appearance of the first suicide case-defining code in the patient’s record.

Aggregated summary data were then securely sent back to the central site. These included prespecified univariate statistics on patient characteristics (eg, age distribution), bivariate analyses of the different risk scores with respect to our outcome of suicidal behavior (ie, odds ratios [ORs] for each concept code), and a summary of the model’s performance, as described in the Results section. Two-sided 95% CIs were calculated for ORs and mean timeliness. The statistical analysis was conducted using R statistical software version 3.5.1 (R Foundation for Statistical Computing). Data analysis was performed from November 2017 to August 2019.

## Results

### Study Population

Of the 3 714 105 patients (2 130 454 female [57.2%]) included in the analysis, 39 162 cases (1.1%) and 3 674 943 noncases (98.9%) from the 5 clinical sites were included in the study. We excluded 10 688 cases because of a lack of data before the index event. Demographic features of the included patients are detailed in Table 1. The 2 major differences across sites were the case prevalence (ranging from 0.40% at BCH to 2.00% at BMC) and the race/ethnicity of the patients (eg, the percentage of African American patients varied from 6.4% at Partners HealthCare to 30.8% at BMC, and the percentage of white patients varied from 36.4% at BMC to 74.7% at WF), reflecting differences in the populations served at each site. Because race/ethnicity is included as 1 of the features of the model, and a separate model was trained at each site, any effects of this difference would be accounted for by the model.

### Risk Factors Across Sites

Each site produced a separate set of model coefficients based on the site-specific patient characteristics and coding patterns and included all available demographic features, diagnostic codes, medications, and laboratory tests. Note that the impact of any given risk factor is a function of its differential frequency between cases and noncases, as well as its overall prevalence. Thus, a rare feature may have a large OR (ie, high partial risk score) but would apply to a smaller fraction of patients compared with a more common feature that might have a more modest effect estimate. All features, regardless of the number of patients for which they were available, were included in the model. A list of patient counts for each risk factor is shown in the eTable in Supplement 1 and the eTable in Supplement 2.

#### Diagnosis and Procedure Codes

As expected, the diagnoses most often associated with suicide attempt were primarily those associated with substance abuse (eg, drug withdrawal syndrome, with ORs of 7.0-12.9) and mental health conditions (eg, borderline personality disorder [ORs, 8.1-12.9] and bipolar disorder [ORs, 0.9-9.1]) (Table 2). In addition, a code for suicidal ideation was associated with a subsequent suicide attempt (mean OR across sites, 11.0; range, 7.6-16.0). With regard to the poisoning by unspecified drug or medicinal substance code, we reviewed 50 EHRs at the Partners HealthCare site and found that for 6 (12%) of the EHRs reviewed, this poisoning code appeared to indicate a prior suicide attempt. However, this code occurred in EHRs for only 0.01% of patients at the Partners site and preceded the case-defining suicide attempt code in only 1.3% of cases. Other diagnoses associated with an increased risk for suicide attempts included traumatic injuries, such as “unarmed fight or brawl,” “assault by unspecified means,” and “observation following alleged rape or seduction” (eTable in Supplement 1). There were several notable site-specific differences; for example, “major depressive affective disorder, recurrent episode, severe, specified as with psychotic behavior” was one of the leading risk factors at BCH (OR, 15.8) but was a weaker predictor at the nonpediatric sites (OR, 3.7; range, 1.73-4.69). The complete table, including 95% CIs for the ORs, can be found in the eTable in Supplement 1.

#### Medications

Medications used to treat mental disorders such as antipsychotics, antidepressants, and mood stabilizers (eg, lithium) were associated with elevated odds of a subsequent suicide attempt (eTable 1 in Supplement 3). At sites that included adult patients, antiretroviral medications used to treat HIV/AIDS (eg, ritonavir) were associated with an increased risk for a suicide attempt. Nicotine replacement therapy also consistently emerged as a leading predictor across the nonpediatric hospitals (OR, 1.3-4.3). In contrast, some medications were associated with risk of suicidal behavior at the children’s hospital (BCH) but not in nonpediatric hospitals, including levonorgestrel with an OR of 1.9 at BCH vs a mean OR of 0.7 at the other sites.

#### Laboratory Tests

Laboratory data were nonuniformly available because the ARCH network common data model ontology only requires that 100 laboratory tests be included. As a result, 3 sites had a limited set of laboratory tests available in their ARCH data sets (eg, BMC with 95 laboratory tests, University of Texas with 171 unique tests, and BCH with 510 unique laboratory tests) (eTable 2 in Supplement 3), whereas Partners and WF had 5814 and 4506 laboratory tests available, respectively. Including only laboratory tests available for at least 100 patients at each of the sites yielded a short list of factors (only 12 basic laboratory tests met this requirement). All available laboratory tests were included in the modeling, regardless of sample size, but for ease of interpretation, we report findings obtained from the sites with sufficient data available for laboratory tests, namely, Partners and WF. At Partners, the most common 20 laboratory tests were all associated with toxicology tests (eg, demoxepam [OR, 24.5], norchlordiazepoxide [OR, 23.3], and phenobarbital [OR, 13.5]). Similarly, at WF, the most common risk factors were also mostly associated with toxicology screening tests (eg, acetaminophen [OR, 13.8] and salicylate [OR, 13.2]), although other tests such as choriogonadotropin-beta (OR, 5.6) and levels of valproic acid (OR, 5.5) were also found to be among the most common risk factors at this site. At BCH, lithium level was the most common laboratory test risk factor (OR, 7.7), although toxicology tests were also associated with subsequent suicide attempt (eg, acetaminophen with an OR of 7.0).

### Model Performance Across Sites

**Figure 2.**
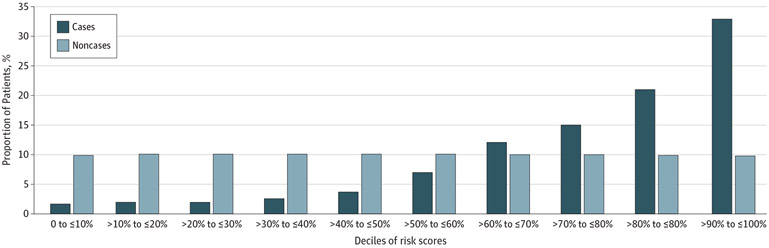
Proportion of Cases by Deciles of Risk Scores Graph shows the percentage of cases and noncases per risk score decile across the 5 included health care systems. The maximal score obtained by each patient was used for calculation.

As shown in Figure 2, 33% of all cases were in the top decile of risk scores (compared with only 10% of the noncases). Across the 5 health care systems, models detected a mean of 38% of cases of suicide attempt with 90% specificity. The eTable in Supplement 2 shows the model performance at each site, measured at 2 different levels of specificity (90% and 99%). Case prevalence in each decile of risk score at individual sites is shown in the eFigure in Supplement 3. The AUCs across validation sites (0.71 [95% CI, 0.70-0.72] to 0.76 [95% CI, 0.75-0.77]) are comparable with that achieved at the original Partners HealthCare site (AUC, 0.73; SE, 0.002). In particular, performance was slightly weaker in the pediatric health system (BCH), with an AUC of 0.72 (SE, 0.01), in contrast to a mean of 0.74 (SE, 0.003) at the other sites. As expected, given the low base rate of suicide attempts, the PPV was low at both 90% specificity (1%-6%) and 99% specificity (3%-14%), whereas negative predictive values were high (98%-100%) (Table 3).

After calculating the overall performance of the model, we assessed the timeliness of the model for predicting suicide attempts. Focusing only on the case patients and using the aforementioned threshold levels, we examined the point which an alert would have been triggered for each of the case patients. Using a high-risk threshold of 90% specificity, the model detected suicide attempts a minimum of 1.3 years (95% CI, 1.1-1.5 years) at BCH and maximum of 3.5 years (95% CI, 3.3-3.6 years) at Partners HealthCare in advance. Across sites, the mean interval between the prediction of high risk and an actual episode of suicidal behavior was 2.1 years at 90% specificity and 1.5 years at 99% specificity. Note that this measure of timeliness indicates the point at which each patient first crossed our specified high-risk threshold, providing an indication of how early elevated risk can be detected. However, each patient’s risk score fluctuates over time. In practice, the model would be recalculated before each encounter according to information available to date. Thus, at a follow-up visit, the accumulation of features that are not associated with increased risk could push a patient’s risk score below the alert threshold. By making this information available to clinicians, the model can be made sensitive in an iterative way to short-term fluctuations in risk.

## Discussion

In this study, we demonstrate that performance of an EHR-based suicide risk prediction strategy is transferable across multiple, diverse health care systems. The present work builds on our prior development and validation of an EHR-based risk prediction model in the Partners HealthCare System using an NBC.^6^ Here we tested the performance of our approach in an expanded data set from Partners HealthCare and 4 additional health care systems. Despite differences in location and patient populations across these systems, model performance was similar (mean AUC, 0.73; range, 0.71-0.76). Not surprisingly, the risk factors with the highest ORs tended to be associated with mental health and substance use conditions. Additional influential predictors included hepatitis C infection, cellulitis or abscess of the hand, rhabdomyolysis, HIV medications, and other features (Table 2, eTable in Supplement 1, and eTable in Supplement 2) that clinicians might be otherwise unlikely to consider in standard suicide risk assessments.

The promising performance of our approach across systems was facilitated by the computational efficiency of NBCs, which allowed sites to easily implement model training and validation on their own data sets. This minimizes the deterioration in performance that might result from porting a single set of model weights across different health care settings. Indeed, as illustrated in Table 2 and eTable 1 in Supplement 3, top predictors in the Partners model had varying effect sizes in the other sites. These differences in effect sizes likely reflect local differences in population health status, prescribing patterns, and billing code assignment in the different systems. In particular, performance was somewhat weaker in the pediatric health system (BCH) with an AUC of 0.72, in contrast to a mean of 0.74 at the other sites.

In recent years, other reports^7,8,18-20^ of EHR-based suicide risk prediction have appeared, using a range of statistical and machine learning methods. For example, Walsh and colleagues^18^ used a random forest approach to train models distinguishing true cases of suicidal behavior among 5167 patients who received an *ICD-9* self-injury code (E95.x). Their model achieved good performance, with AUCs of 0.80 to 0.84 and PPVs of 0.74 to 0.79 over time frames of 7 to 720 days before a documented suicide attempt.^18^ As noted, however, this model was trained to identify cases among the subset of individuals with self-injury diagnoses rather than cases within the general population seeking care across the entire health care system. Simon et al^7^ used penalized lasso regression to develop a risk prediction model for suicidal behavior (attempts and death) in 2.9 million patients seen in primary care or mental health settings across 7 health systems participating in the Mental Health Research Network. Their model focused on prediction of suicidal behavior in the 90 days following a clinical visit and was trained using 313 clinical characteristics comprising demographic and EHR data (drawn from up to 5 years before the index visit) and results obtained from the Patient Health Questionnaire-9 measure of depression. Models calculated for suicide attempts and death achieved AUCs of 0.83 to 0.86, and PPVs for suicide attempt were comparable to those observed in our study (PPV, 3.6% at 90% specificity and 10.4% at 99% specificity).^7^ The model developed by Simon and colleagues^7^ has several notable differences from ours. These include the smaller number of predictors used to train their models, limiting the prediction window to 90 days following an outpatient visit tied to an *ICD-9* diagnosis of a mental health condition, inclusion of a standardized depression measure (Patient Health Questionnaire-9), and restricting the patient population to those insured by the health system’s insurance plan. In addition, their model incorporated prior suicide attempts as a predictor, whereas ours was restricted to the first documented occurrence of suicidal behavior. Overall, the range of methods and clinical settings covered by prior studies and the present study demonstrates the potential value of a variety of approaches for EHR-based risk prediction of suicidal behavior. This has become a fertile area of research, and future studies will be needed to clarify the relative utility of different statistical and machine learning methods.

### Strengths and Limitations

This study has several strengths. The NBC approach is computationally efficient and allows scalable application to multiple health systems that may vary in their case prevalence, EHR coding practices, and population-specific distributions of risk predictors. In contrast to other reported efforts, this approach also incorporates contributions from the full range of phenotypic data (approximately 30 000 features) available in the EHR and produces readily interpretable ORs for each predictor. Furthermore, unlike most other efforts to leverage EHR data for prediction of suicidal behavior, we derived our model in the full patient population in each health care system rather than conditioning on specific settings or prior diagnoses. As such, our model should be broadly applicable in health system data.

The results are also subject to several limitations. First, the NBC approach is only one of many possible modeling methods; alternative machine-learning methods (eg, random forest or deeplearning neural networks) may yield similar or better performance but can often result in “black box” models that are less interpretable and more challenging to compute. Second, as noted, our model is designed to predict the first documented occurrence of suicidal behavior; as such, prior suicide attempts, a known risk factor, are not available to the model. We chose this approach because it can be difficult to determine whether multiple suicide-related codes that occur within a short period in a patient’s record are actually distinct occurrences or part of the same suicidal episode. Indeed, 1 influential predictor in our models, poisoning by unspecified drug or medicinal substance (Table 2), might indicate a prior suicide attempt. To explore this, we reviewed 50 EHRs at the Partners HealthCare site and found that for 6 (12%) of the EHRs reviewed, this poisoning code appeared to indicate a prior suicide attempt. However, this code occurred for only 0.01% of patients at the Partners site and preceded the case-defining suicide attempt code in only 1.3% of cases. Thus, although this feature had a large effect size, it was rare. Nevertheless, this illustrates the point that any case definition based on real-world EHR data will have limitations.

Third, we restricted our model to structured EHR data because they are readily available in EHR systems across sites, facilitating implementation of our approach across health systems. However, *ICD-9* code data have limitations (as we have previously discussed).^6^ We and others have shown that text-mining using natural language processing can enhance the performance of EHR-based phenotyping.^21-23^ For example, we recently showed that natural language processing substantially improved the detection of suicidal behavior among pregnant women.^24,25^ We are currently exploring the addition of natural language processing to our suicide risk prediction models. Fourth, because patients may receive care outside the participating health care system, relevant predictors or outcomes may have been missed, resulting in weaker than optimal performance of our models. In addition, although the AUC values were generally good across sites, the PPVs were low. This was expected because PPV depends strongly on the prevalence of the outcome, and suicide attempts are low base-rate events. Nevertheless, this means that most patients who exceed the alert threshold would not be expected to engage in suicidal behavior.

Recent reviews^26,27^ of suicide prediction models concluded that the low PPV of existing models hinders their utility in clinical settings. However, PPV alone is not necessarily the criterion for evaluating the utility of a prediction algorithm. The threshold for acceptable PPV depends on the intent of a risk prediction model. Rather than relying on such algorithms as a stand-alone method for determining absolute risk and clinical disposition, we view suicide risk prediction algorithms as a useful tool for stratifying individuals at the higher end of a risk distribution and assisting clinicians in focusing on such individuals for further evaluation. It is important to consider the context in which such algorithms might be deployed. The incidence of suicide attempts and deaths is increasing, and, at present, clinicians do no better than chance at predicting suicidal behavior.^5^ An algorithm that enhances that performance by several fold and is used to flag the need for more systematic evaluation may, nonetheless, provide a cost-effective option. As Simon and colleagues^28^ have recently noted, the PPVs associated with available EHR algorithms such as ours are comparable with those recommended for guiding interventions to prevent breast cancer and cardiovascular disease. Nevertheless, enhancing PPV is a worthy goal. A number of strategies could be pursued to enhance the model PPV, including training models in subpopulations (eg, patients seen in psychiatric services) and incorporating natural language processing or insurance claims data.

We emphasize that our model is designed to inform and not replace clinical judgment. We envision our model as a first-stage screening aid that might alert clinicians to a patient’s heightened risk profile and the possible need for more targeted evaluation and risk assessment. The computational efficiency of the NBC method could enable risk calculations and clinical decision support at the point of care (eg, using a SMART [Substitutable Medical Apps, Reusable Technology] application embedded in the EHR interface).^29^ Implementation of risk alerts would also need to be accompanied by effective options for managing patients found to be at increased risk; these might include recommendations for more in-depth risk assessment and follow-up monitoring.

## Conclusions

This study validates an EHR-based approach to predicting suicidal behavior across 5 independent and geographically dispersed US health care systems. Suicide prediction in diverse clinical settings may benefit from disseminating a robust modeling process (as done here), rather than transferring a specific predictive model. Our approach demonstrates the feasibility of developing scalable, interpretable risk prediction algorithms using real-world health care data.

## Supplementary Material

Appendix 1 SUPPLEMENT 1.
**eTable.** All ICD diagnoses With Associated Odds-Ratios, by Site

Appendix 2 SUPPLEMENT 2.
**eTable.** Complete List of Medications With Odds-Ratio, by Site

## Figures and Tables

**Table 1.** Demographic Description and Case Distribution in the 5 Sites Included in the Study

	Patients, No. (%)				
Variable	Partners HealthCare (n = 1 931 672)	BMC (n = 477 447)	WF (n = 528 110)	UT (n = 501 486)	BCH (n = 286 078)
Case prevalence	25 730 (1.30)	9550 (2.00)	8967 (1.70)	4462 (0.89)	1141 (0.40)
Included cases	20 466 (1.10)	8026 (1.68)	6924 (1.31)	2778 (0.55)	968 (0.34)
Years of data	17	17	10	13	8
Follow-up duration, median (IQR), y	4.8 (1.25-10.3)	4.2 (1.1-9.4)	4 (1.2-7.3)	3.7 (1.0-8.1)	2.7 (0.8-5.7)
Sex					
Female	1 122 221 (58.1)	255 403 (53.5)	298 355 (56.5)	299 266 (59.7)	155 209 (54.3)
Male	809 276 (41.9)	222 038 (46.5)	229 740 (43.5)	201 791 (40.2)	130 850 (45.7)
Race/ethnicity					
African American	123 903 (6.4)	147 268 (30.8)	92 269 (17.5)	99 821 (19.9)	21 656 (7.6)
American Indian, Eskimo, or Aleut	NA	1985 (0.4)	1501 (0.3)	1640 (0.3)	436 (0.2)
Asian	71470 (3.7)	21 020 (4.4)	5476 (1.0)	NA	7442 (2.6)
White	1 404 699 (72.7)	173 808 (36.4)	394 454(74.7)	212 733 (42.4)	165 052 (57.7)
Hispanic or Latino	133 411 (6.9)	46 554 (9.8)	299 (0.1)	40 795 (8.1)	30 217 (10.6)
Other or unknown	198 189 (10.3)	86 812 (18.2)	34 111 (6.5)	146 497 (29.2)	91 492 (32.0)

Abbreviations: BCH, Boston Children’s Hospital; BMC, Boston Medical Center; IQR, interquartile range; NA, not applicable; UT, The University ofTexas Health ScienceCenterat Houston; WF, Wake Forest Medical Center.

**Table 2.** Top 20 Diagnostic Codes Associated With an Increased Risk of Suicidal Behavior by Case Prevalence Across the Sites^a^

*ICD-9* Code	Diagnosis	OR					Cases, No. (%)	Noncases, No.
		Partners HealthCare	BMC	WF	UT	BCH		
9779	Poisoning by unspecified drug or medicinal substance	14.1	9.1	12.7	22.3	28.3	252 (12.8)	1711
2920	Drug withdrawal syndrome	12.9	10.2	7.0	9.4	NA^b^	541 (11.7)	4070
30420	Cocaine dependence, unspecified use	11.2	7.9	16.4	4.9	7.7	315 (10.6)	2667
29284	Drug-induced organic affective syndrome	11.0	5.0	17.1	8.1	NA^b^	152 (10.5)	1289
V6284	Suicidal ideation	11.5	7.6	10.1	9.7	16.0	1323 (10.4)	11 424
30183	Borderline personality	9.8	8.1	12.9	9.7	NA^b^	289 (9.9)	2645
30590	Other, mixed, or unspecified drug abuse, unspecified use	13.2	6.9	7.9	4.6	10.2	1757 (9.8)	16 205
309	Unspecified neurotic disorder	11.1	6.9	6.0	14.9	1.6	686 (9.6)	6461
3030	Acute alcoholic intoxication in alcoholism, unspecified drinking behavior	11.6	4.8	8.2	8.3	13.9	424 (9.6)	4002
30560	Cocaine abuse, unspecified use	12.1	6.8	8.5	4.6	3.3	1019 (9.6)	9642
8840	Multiple and unspecified open wound of upper limb, without mention of complication	8.3	6.4	10.7	28.5	28.3	225 (9.4)	2166
3040	Opioid type dependence, unspecified use	10.5	5.7	4.5	6.6	4.4	1038 (8.7)	10 834
8803	Open wound of upper arm, without mention of complication	8.9	4.0	8.1	20.0	12.7	76 (8.6)	804
30490	Unspecified drug dependence, unspecified use	9.1	6.6	4.6	6.3	8.6	448 (8.3)	4920
8822	Open wound of hand except fingers alone, with tendon involvement	8.3	7.0	6.0	9.4	22.5	142 (8.1)	1615
E966	Assault by cutting and piercing instrument	8.0	4.3	8.9	7.5	0.1	215 (7.6)	2614
30501	Alcohol abuse, continuous drinking behavior	6.6	6.4	4.3	3.4	NA^b^	776 (7.6)	9440
29650	Bipolar affective disorder, depressed, unspecified degree	7.5	5.2	6.9	0.9	9.1	520 (7.2)	6675
3019	Unspecified personality disorder	7.5	3.1	9.2	5.2	NA^b^	222 (7.1)	2921
29660	Bipolar affective disorder, mixed, unspecified degree	7.1	6.3	6.4	1.6	NA^b^	312 (7.0)	4115

Abbreviations: BCH, Boston Children’s Hospital; BMC, Boston Medical Center; *ICD-9, International Classification of Diseases, Ninth Revision;* NA, not applicable; OR, odds ratio; UT, The University of Texas Health Science Center at Houston; WF, Wake Forest Medical Center.

a Table shows only diagnoses available for at least 100 patients (cases and noncases combined) in each of the nonpediatric sites. The 95% CIs for the ORs are shown in the eTable in Supplement 1.

b The ORs for diagnoses with fewer than 10 case patients were omitted.

**Table 3.** Model Performance Metrics and Prediction Time for 5 Sites, by Specificity^a^

Specificity level, site	Case prevalence, No. (%)^b^	Percentage			AUC (SE)	Prediction time, mean (95% CI), y before suicide attempt^c^
		Sensitivity	PPV	NPV		
99% Specificity						
Partners	10 199 (1.0)	8	8	99	0.73 (0.002)	2.5 (2.3-2.7)
BMC	4013 (1.7)	9	14	98	0.74 (0.003)	1.9 (1.7–2.1)
WF	3462 (1.3)	7	9	99	0.71 (0.004)	1.6 (1.4-1.8)
UT	1376 (0.6)	8	4	99	0.76 (0.005)	0.3 (0.1-0.5)
BCH	466 (0.34)	11	3	100	0.72 (0.01)	1.0 (0.7-1.3)
90% Specificity						
Partners	10 199 (1.0)	36	4	99	0.73 (0.002)	3.5 (3.3-3.6)
BMC	4013 (1.7)	39	6	99	0.74 (0.003)	2.4 (2.3-2.5)
WF	3462 (1.3)	33	4	99	0.71 (0.004)	1.9 (1.8-2.0)
UT	1376 (0.6)	39	2	100	0.76 (0.005)	1.4 (1.2-1.6)
BCH	466 (0.34)	37	1	100	0.72 (0.01)	1.3 (1.1-1.5)

Abbreviations: AUC, area under the receiver operating curve; BCH, Boston Children’s Hospital; BMC, Boston Medical Center; NPV, negative predictive value; PPV, positive predictive value; UT, The University of Texas Health Science Center at Houston; WF, Wake Forest Medical Center.

a Results, including the case prevalence, are all based only on the 50% validation set of each of the sites.

b Case prevalence represents the number of cases and their percentage in the 50% randomly selected validation set at each of the sites.

c Prediction time is the interval between prediction of high risk and first documented episode of suicidal behavior.

## Key Points

### Question

Can a process for training machine-learning algorithms based on electronic health records identify individuals at increased risk of suicide attempts across independent health care systems?

### Findings

In this prognostic study, using a supervised learning approach applied to structured electronic health record data from more than 3.7 million patients across 5 diverse US health care systems, models detected a mean of 38% of cases of suicide attempt with 90% specificity a mean of 2.1 years in advance.

### Meaning

These findings suggest that a computationally efficient machine-learning approach leveraging the full spectrum of structured electronic health record data may be able to detect the risk of suicidal behavior in unselected patients and may facilitate the development of clinical decision support tools that inform risk reduction interventions.
